# Taking intestinal microbes and the immune system as opportunities to maintain the intestinal health of piglets: review

**DOI:** 10.5713/ab.25.0100

**Published:** 2025-05-19

**Authors:** Xiaohong Hou, Yue Chen, Fengming Chen, Yulong Yin, Kang Xu

**Affiliations:** 1 Hunan Provincial Key Laboratory of Animal Nutritional Physiology and Metabolic Process, National Engineering Laboratory for Pollution Control and Waste Utilization in Livestock and Poultry Production, Institute of Subtropical Agriculture, Chinese Academy of Sciences, Changsha, China; 2 College of Veterinary Medicine, Henan Agricultural University, Zhengzhou, China; 3 Hunan Provincial Key Laboratory of the Traditional Chinese Medicine Agricultural Biogenomics, Changsha Medical University, Changsha, China

**Keywords:** Gut Microbes, Immunomodulation, Intestinal Health, Microbial Regulation

## Abstract

Numerous commensal microbes colonize in the pig gut, while the microbiota gets benefits from the gut environment, it additionally assists significantly with the host’s development, metabolism, and immunity. The immune system can regulate the homeostasis of gut microorganisms by secreting a variety of effector factors. Similarly, gut microorganisms can also regulate the intestinal immune system via specific components or metabolites. Despite their close relationship, microbes and immune cells have their own individual ecological niche in the gut. Microbes are confined to the intestinal lumen, while immune cells are located in the intestinal tissues, and the mechanisms of how they interact with each other to promote intestinal health have not yet been thoroughly investigated. This article focuses on the different mechanisms by the interaction between intestinal immune system, gut microorganisms and microbial metabolites to regulate the intestinal health of piglets, and discusses the strategies to enhance the intestinal health of piglets through dietary interventions in order to provide theoretical support promoting the healthy growth of piglets.

## INTRODUCTION

Gut microbes play a significant role in influencing the physiological state of the body through complex interactions. A stable microecosystem is critical for intestinal physiological and biochemical processes [1]. However, environmental factors and internal dysfunction can destroy this stability and balance, leading to immune dysregulation and impaired intestinal barrier function. Currently, the gut microbes have been shown to have a significant impact on the regulation of immune systems [2].

Complex interactions exist among intestinal microflora, intestinal epithelium and mucosal immune system. Symbiotic microorganisms in the gut can stimulate the proliferation of lymphocytes through their antigens to promote the development of body resistance, and can also modulate host physiology by producing diverse metabolites. For instance, *Lactobacillus* in the intestine can use Trp to promote their proliferation and produce indole. Indole, an aromatic hydrocarbon receptor (AHR) ligand, can activate the AHR-interleukin (IL) 22 axis to balance the mucosal response and maintain intestinal health [3]. Similarly, intestine cells can regulate flora and immunity by secreting active substances. Intestinal cells generate intestinal alkaline phosphatase (IAP), which is essential for preventing intestinal inflammation, regulating gut flora distribution, and inhibiting bacterial translocation [4]. Under stress conditions, including weaning, the production of IAP in pigs will be significantly inhibited, which is the reason for the occurrence of many different post-weaning diseases and the increased sensitivity to intestinal infection [5]. In fact, the complex interactions among nutrients, intestinal immunity and the microflora are the most important elements influencing the intestinal health of piglets. Identifying the critical targets for controlling intestinal health requires an in-depth comprehension of the impact of the gut microbes and immune system. With the goal of providing a theoretical foundation for the development of more sensible and successful methods of gut health regulation in the future, this study discusses some of the current approaches to gut health regulation and gives a thorough overview of the effects of intestinal immunity, gut microbes, and their metabolites on the intestinal health of piglets.

## THE INTESTINAL IMMUNE SYSTEM AND GUT HEALTH OF PIGLETS

Piglets’ intestinal immune system is not fully mature at birth, and continues to develop during the perinatal period, and it reaches maturity at five to seven weeks after birth [6]. Three to eight weeks after birth is the critical period for T cell development in the piglets’ intestine [7]. During this period, piglets are particularly vulnerable to infections due to their immature intestinal immune systems [8]. Most piglets are weaned at a stage where their intestinal immune systems have not yet fully matured, which is an important reason why diarrhea occurs in piglets after weaning and further leads to high morbidity and mortality. The intestinal mucosa and mucus layer are essential components of intestinal immunity that regulate piglet intestinal health.

### Intestinal mucosa and gut health

**Figure 1:**
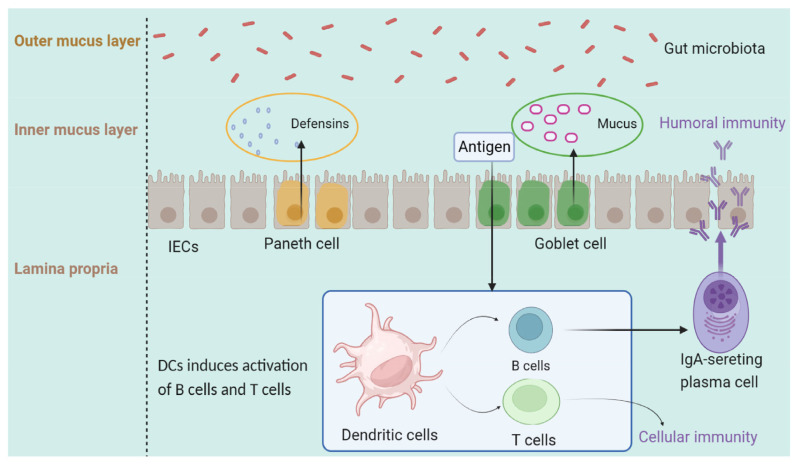
Intestinal mucosal immune system. Mucus produced by goblet cells serves to restrict microbial translocation by reducing direct contact between microorganisms and intestinal epithelial cells (IEC). Antimicrobial proteins such as defensins can be secreted by intestinal and goblet cells. Some bacterial antigens penetrating mucosal layer can be absorbed by dendritic cells (DCs) in the lamina propria of intestine. Immunoglobulin-A (IgA) is secreted into the intestinal cavity by plasma cells, which develop from B lymphocytes stimulated by DCs.

The intestinal mucosa is the main physical barrier to prevent bacteria and toxins from entering the digestive system, and is critically important for maintaining intestinal health. The mucosal immune system contains the largest reservoir of immune cells, which can quickly activate the immune response and protect the body from pathogenic infection [9]. Intestinal epithelial cells (IEC), Peyer’s patches (PP) and intraepithelial lymphocytes (IEL) are the main members of the intestinal mucosal immune system (Figure 1).

### Intestinal epithelial cells and gut health

As part of the intestinal barrier, IEC can isolate the host from commensal bacteria to maintain intestinal homeostasis. In addition, IEC is essential for activating immune response [10]. The antimicrobial peptides (AMPs) secreted by IEC in piglets can regulate the immune system and enhance resistance to pathogenic bacteria [11]. Porcine beta-defensin (PBD) 2 is a type of AMP that may alleviate mucosal damage and inflammation caused by colitis, and the nuclear factor-kappa B (NF-κB) pathway activated by Toll-like receptors (TLRs) can quickly boost PBD114 expression [12], which helps to regulate the homeostasis of intestinal epithelium in piglets. Within IEC, the signaling pathway mediated by NF-κB functions as a frontline sensor for contact with microorganisms, orchestrating immune homeostasis through balanced regulation, whereas its pathological overactivation initiates pro-inflammatory cascades that compromise tissue integrity. Porcine butyrophilin-like 5, which is present in the IEC, prevents p65-mediated NF-κB pathway activation [13].

Due to this particular position of the IEC at the crossroads of mediating the interactions between the gut microbe and the intestinal immune cells, this exchange between them has a far-reaching effect on the host’s overall health.

### Intraepithelial lymphocytes and gut health

A group of lymphocytes residing within IEC, termed IEL, actively participate in intestinal barrier formation [14]. These lymphocytes are highly mobile, and active in the space between the epithelial layer and the basement membrane and its position is between adjacent epithelial cells in the lateral intercellular space and along the basal surface [15]. This positioning and movement mode allows them to start the immune response in time when the intestinal tract is infected by pathogens. Through mucosal vaccination, lymphocytes can create protective adaptive immunity [16], while some lymphocytes can provide immune protection against many bacteria through innate immune memory [17].

The intraepithelial compartment comprises two principal lymphocyte populations: intraepithelial T lymphocytes (T-IEL) and intraepithelial innate lymphoid cells (ILC). In the intestine of four to eight weeks old pigs, the amount of T-IEL increases with age, and these T cells have been proven to be of the αβ and γδ T cell lineages. As pigs get older, the composition of T-IEL differed between intestinal sites, with CD2CD8α γδ T cells and CD4^++−^CD8α αβ T cells making up about 78% of total T-IEL [7], however, functional significance that they represent needs further investigation.

Despite lacking T cell receptors, ILC mediate adaptive immune responses through mechanisms analogous to effector T cells [18]. The ILC family is categorized into three functional groups (group 1, group 2 and group 3), and their development requires the transcription factors inhibitor DNA binding 2 and nuclear factor IL-3 regulated [14,19]. Due to the lack of clear labelling and identification methods, there are few studies on pig intestinal ILCs. In order to identify the role of ILCs in the pig intestine, Wiarda et al [20] utilized single cell RNA sequencing to determine the gene expression profile and location background of the ILC1 and ILC3 in pig ileum. They found that the function of ILCs in pig ileum is similar to that in human and mouse intestines, such as the monitoring function of ILC1 in epithelium, and the role of ILC3 in immune defence, regulating microbial and gut associated lymphoid tissue development, and maintaining tissue homeostasis.

In piglets, IEL immune activation is usually related to increases in the number of IEL [21]. The recruitment and development of IEL are significantly influenced by the microbiota, and microbiota-influencing dietary supplements, which include probiotics and prebiotics, have an immune-modulatory effect on intestinal IEL in pigs [22,23]. Overall, IEL are a type of cell with plasticity potential. Intestinal environmental factors, including gut bacteria and their by-products, shape the immunological characteristics of IEL.

### Peyer’s patch and gut health

Aggregations of lymphoid follicles within the intestinal mucosa, termed PP, constitute critical components of intestinal mucosal immunity. These organized lymphoid follicles function as immunological sensors of the intestine due to the fact that they transport luminal antigens and microorganisms into lymphoid tissues within the intestinal mucosa [24]. The small intestine of pigs has two types of PP. The PP in the lower portion of the ileum is known as the ileal PP (IPP), and it is made up of a series of closely grouped follicles rich in B cells, whereas the follicles in the jejunum PP (JPP) are independent.

The organogenesis of both JPP and IPP can be modulated separately in pigs. Following gut microbial colonization, the follicular architecture demonstrates enhanced maturation in JPP aggregates compared to IPP, which maintain structural stability throughout postnatal development [25]. Upon maturation, JPP begin to exert an immune role, producing a variety of immunoglobulin A (IgA) with the help of T cells and are critical for mucosal immunity [26,27]. Pigs’ IPP have distinctive characteristics that are not observed in other animals. Between the 76th and 91st day of embryo, the organogenesis of IPP started, and the expression of chemokine C-X-C motif ligand 13 and chemokine C-C motif ligand 19 increased, which is important for the recruitment of B cells and T cells in lymphoid tissues. After being born, IPP began to develop rapidly [28]. These findings indicate that particular alterations in pigs’ early intestinal environments may be connected to the peculiarities of IPP in pigs. In the absence of T cell help, the IPP of pigs seem to be the sites of the initial immune response that generates undiversified IgA. The binding of IL-21 to its receptor on B cells induces phosphorylation of Janus kinase 1 (JAK1) and signal transducer and activator of transcription (STAT) 1 and STAT3. This activation of the JAK-STAT signaling pathway promotes plasma cell differentiation, ultimately enhancing secretory immunoglobulin A (sIgA) production [29]. This also serves as a reminder that stimulating PP to secrete antibodies by mucosal immunity is one of the tactics for protecting piglets. Furthermore, it aids in the growth and well-being of young pigs by facilitating their adaptation to abrupt and noteworthy modifications in their intestinal microbiome.

## INTESTINAL MUCUS LAYER AND GUT HEALTH

The mucus layer, produced by goblet cells, is composed of an inner and outer layer, and serves as a physical barrier to protect the intestinal lumen from bacteria or other antigenic substances. The inner layer adheres to the epithelial cell to prevent bacterial invasion and contains AMPs, IgA and other molecules. The outer mucus layer is loose, which provides a habitat for microorganisms [30].

Intestinal mucus is primarily a reticular polymeric structure composed of highly O-glycosylated mucin [31]. In addition to providing certain bacteria with nutrients, these mucin-derived O-glycans safeguard the mucus layer against degradation by bacterial proteases [32]. Additionally, the polysaccharide structure of mucin provides binding sites for intestinal bacteria through its adhesive components [33]. Diarrhea in piglets will destroy the intestinal mucus layer, induce bacterial translocation and disrupt the intestinal microenvironment, making them more vulnerable to inflammation. The activation of inflammatory corpuscles of nucleotide oligomerization domain (NOD)-like receptor protein 3 (NLRP3) in the colon of piglets with diarrhea is the primary cause of this condition, which leads to the release of pro-inflammatory cytokines, ultimately lead to intestinal inflammation [34]. Activated NLRP3 inflammatory vesicles can help combat certain pathogens, while excessive activation can lead to harmful inflammation. Mucin 2 (MUC2) and fraction III in pig intestinal mucin have strong antiviral activity, and calpain-1 (a new antiviral protein found in porcine intestinal mucus) produced by goblet cells has made a significant contribution to the antiviral activity of fraction III. Calpain-1, a calcium-activated cysteine protease, can bind and hydrolyse the S1 structural domain of viral proteins to inhibit viral invasion [35].

More research is showing most pathogens invade and infect the gut mucosa as their primary site of infection. One of the key strategies for preserving intestinal health is to strengthen the integrity of the intestinal mucus barrier. Although controlling intestinal immunity makes sense for improving piglets’ intestinal health, understanding the complex workings of the intestinal immune system is somewhat difficult. The presence of diverse immune cells within the intestine complicates the determination of their interactions and overall function. Determining the functions of various intestinal immune cells is the first step towards better understanding intestinal immunodynamics and their impact on intestinal health.

## INTERACTION BETWEEN THE INTESTINAL MICROBIOTA AND IMMUNE SYSTEM

Intestinal microbial community consists of trillions of microorganisms, forming a mutually beneficial symbiotic connection with the host. Microbial colonization is an important factor affecting intestinal development. Germ-free (GF) animals’ intestinal immunity and barrier are not fully established due to the lack of microbial colonization, making them more susceptible to intestinal inflammation.

There are some gut bacteria that interact with the host through pattern recognition receptors (PRRs). When pathogenic microorganisms enter the body, PRRs identify pathogen-associated molecular patterns and activate signaling transduction downstream, thereby stimulating the innate immunity [36]. Through PRRs, gut microorganisms can regulate the level of inflammatory-related genes and AMPs, and in turn, PRRs expression can also influence the gut microbiota in healthy or diseased conditions [37]. Different studies have confirmed the crucial role of gut microbial colonization on the evolution of PRRs.

Piglets show different expression patterns of TLRs in different intestines, with increased expression of TLR1, 2, 4, and 9 in the jejunum during the first week of life, whereas in the cecum, TLR9 is stably expressed at different time points, which may be associated with the different species of microorganisms in different intestinal segments [38,39]. Disruption of microbial communities in the gut can adversely affect the colonic epithelium. In newborn piglets, the destruction of microflora is characterized by the decrease of Proteobacteria and Fusobacteriota will significantly alter the epithelial function by reducing the intestinal epithelium’s innate immunological defense. During this process, the expression levels of lipopolysaccharide (LPS) binding protein and LPS sensors TLR4 in the colon epithelium of piglets were significantly reduced, and colon epithelial stem cells were destroyed [40]. When weaned piglets were exposed to plant-oriented microbiome, the expressions of TLR, NOD1 and cytokines in the jejunum increased, thereby activating intestinal defence and reducing mucosal permeability [41]. Besides PRRs, gut microbes are crucial for immunoglobulin synthesis. GF animals exhibit severely diminished IgA production by B cells residing in PP and lamina propria (LP), a deficit that is rapidly rectified upon microbial colonization [42,43]. Gut symbiotic bacteria may collaborate with dendritic cells (DCs) and IEC to promote the generation of IgA [44]. After application of *Lactobacillus rhamnosus* GG (LGG) isolated from healthy gut to newborn piglets, it was found that LGG could promote IgA production in LP, influence the composition of the Ig CDR3 region of B cells and promote B cell development. Exploration of the mechanism revealed that p40 protein from LGG could activate the epidermal growth factor receptor/protein kinase B (AKT) and NF-κB signaling pathways, and stimulate the B cells’ production of IgA [45]. Similarly, IgA can also regulate the colonization of intestinal microorganisms. The IgA response mediated by CD138 plasma cells can regulate the symbiotic relationship between *Bacteroides uniformis* (*B. uniformis*) and its host in the weaning period, which is very important for *B. uniformis* to occupy the intestinal niche. Furthermore, the lack of IgA caused a disruption in the intestinal niche occupied by *B. uniformis*, exacerbating intestinal inflammation in both weaned piglets and IgA-deficient mice [46]. By comparing the phenotype-transcriptome changes in intestinal PP between specific pathogen-free piglets and GF piglets, it was found that lack of symbiotic microflora would lead to PP hypoplasia in piglets, and that the microflora could significantly modulate B cell function [47].

Numerous studies have investigated the effects of gut microbes on piglets’ immune systems, but further surveys are required to pinpoint the precise relationships between gut immunity and the microbiota. An in-depth analysis of these mechanisms is crucial for the selection of valid interventions that will improve the intestinal health of pigs. Obviously, research targeting microorganisms will remain the main focus. Utilizing in vitro models, such as organoids, can facilitate the study of these interactions.

## EFFECTS OF GUT MICROBES ON INTESTINAL HEALTH

A mutually beneficial symbiotic relationship exists between the gut flora and the host. In particular, microflora-induced signals can promote piglet health by influencing host physiology, immune response, and intestinal barrier function [48].

Piglets gut microbiota develops gradually from birth to weaning until it stabilizes. During the first 12 hours after birth, *Clostridium* was the dominant flora in the gut of piglets, but it was quickly replaced by *Streptococcus* within one day. At the ages of 5, 10 and 20 days, Lactobacillaceae with anti-inflammatory and antioxidant effects became the dominant flora [49,50]. Studies on the structure and changes of gut microorganisms in weaned piglets have shown a gradual increase in the Firmicutes and Bacteroidetes with age, while the relative abundances of Fusobacteria and Proteobacteria gradually decreased. However, the abundance of Actinobacteria did not change significantly [51]. After the weaning process, piglets undergo a significant transition from breastfeeding to consuming more complex solid feeds. The piglets’ gut microbiome underwent a dramatic shift in composition, with a decrease of *Lactobacillus* spp., and a marked rise significant in *Clostridium* spp. and *Escherichia coli* (*E. coli*) [52]. This period of transition renders piglets vulnerable to infections caused by external pathogenic bacteria. As piglets acclimate to these dietary changes, the gut microbiota, primarily comprised of anaerobic bacteria, forms a diverse ecological community [53]. *Bacteroides*, as the dominating genus in the gut of piglets before weaning, assists piglets metabolize milk-derived carbohydrates because of their ability to utilize multiple saccharides [54]. Similarly, piglets can adapt to diet changes with the help of *Prevotella*, which can metabolize complex dietary polysaccharides, and it is also the main producer of short-chain fatty acids (SCFAs), which regulate host energy metabolism and gut health [55]. *Bacteroides* and *Prevotella* are regarded as crucial factors in maintaining gut health in weaned piglets. While there are numerous other microbiota that are also beneficial to piglet gut health, deeper research is essential to fully elucidate the vast potential of gut microorganisms.

## EFFECTS OF GUT MICROBES METABOLITES ON INTESTINAL HEALTH

**Figure 2:**
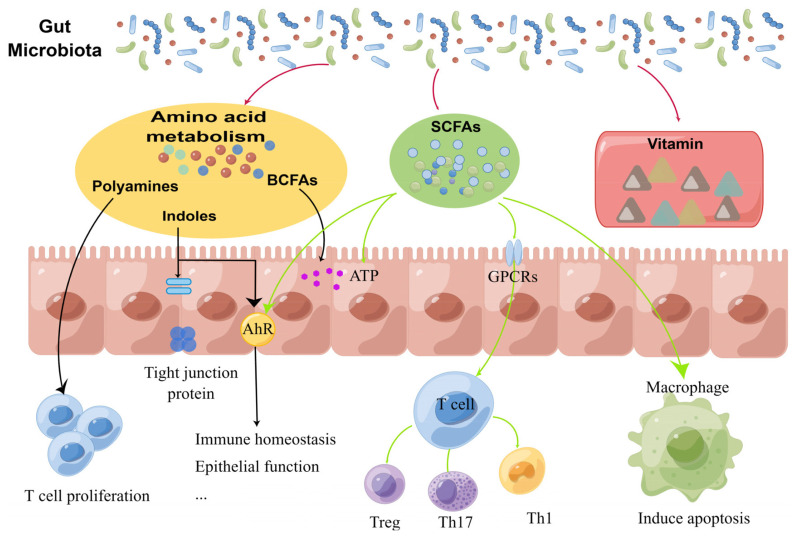
Microbiome-derived metabolites and their effects on gut health. The gut microbiota will produce various metabolites, such as short-chain fatty acids (SCFAs), amino acid metabolites (polyamines, indoles, and branched-chain fatty acid [BCFAs]), and vitamins. Intestinal immune function, metabolism, and homeostasis are all regulated by these metabolites.

Metabolites produced by microorganisms are important intermediate products of the interactions between hosts and microorganisms, which can prevent inflammation, modulate intestinal immunity and maintain intestinal barrier integrity [56,57]. The physiological activity of the body is regulated by gut microbes and their metabolites, which positively or negatively affect host health through a variety of associated signaling pathways (Figure 2).

### Indole metabolites

Indole and its derivatives are primarily synthesized through microbial fermentation of Trp. Within the intestinal barrier system, signaling through the AHR pathway is recognized as a pivotal regulator of immune responses. It has been discovered that indole and its derivatives activate the AHR in IEC, thus improving the mucosal barrier of the intestine and increasing the generation of tight junction proteins [3], and reduce inflammation via activation of the pregnane X receptor [58]. *Lactobacillus* and *Clostridium* in gut are associated with Trp metabolism, and *Lactobacillus* is particularly abundant in the intestinal of the Min pigs, which is known for its resistance to disease, and this is the key reason for the indole derivatives’ high levels in the intestinal of the Min pigs [59]. Indole propionic acid (IPA) is an indole metabolite produced by intestinal commensal microbiota through deamination, which can increase the production of tight junction proteins, thus improving intestinal barrier function. It also serves on certain tissues via blood circulation to maintain systemic homeostasis [60]. The intestinal barrier’s function is intricately connected to immunological activation. In LPS-stimulated Caco-2 or HT29 cells, IPA treatment significantly inhibited activation of the PI3K/AKT/mechanistic target of rapamycin (mTOR) signaling pathway and downregulated inflammatory cytokine expression [61]. Additionally, IPA also significantly decreased the elevated expression of interferon-γ, tumor necrosis factor-α and IL-1β induced by dextran sulfate sodium in mice and alleviated intestinal inflammation by activating AHR [62]. Indole-3-carboxaldehyde, a derivative of indole from bacterial catabolism of metabolised Trp, regulates intestinal homeostasis by accelerating intestinal epithelial proliferation without affecting intestinal morphology and permeability [63]. When the intestinal epithelium is damaged and inflammation occurs in piglets, the increase of Trp metabolite indole-3-acetic acid (IAA) in the colon contents will alleviate intestinal injury [64]. In weaned piglets, increased levels of indole and IAA produced by the colon microbiome correlated with an increase in AHR and tight junction proteins [65]. These evidences show that indole derivatives have the potential to regulate intestinal health. Indole, as a new signal molecule, plays an important role in safeguarding the stability of the intestinal environment. In-depth study on the generation and mechanism of indole derivatives will enable us to find effective strategies to enhance intestinal health.

### Short-chain fatty acids

**Figure 3:**
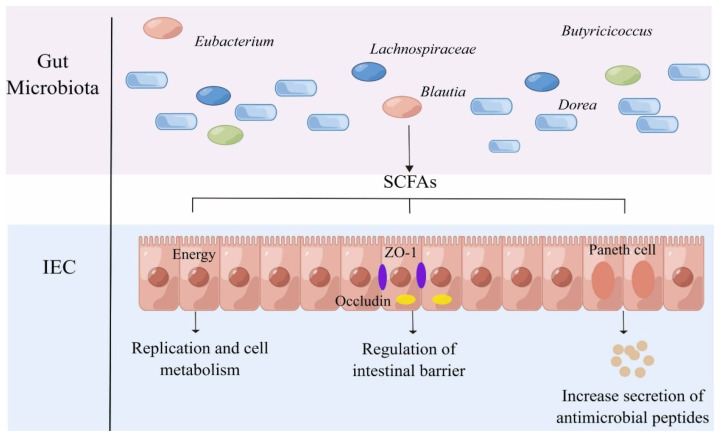
Microbiome-derived short-chain fatty acids (SCFAs) metabolites and their effects on intestinal epithelial cell (IEC). SCFAs produced by the gut microbiota (mainly Blautia, Eubacterium, Dorea, Butyricicoccus, and Lachnospiraceae, etc.) can provide energy for the replication and metabolism of IEC and can regulate the function of intestinal barrier by affecting the generation of tight junction proteins in IEC. Moreover, SCFAs promote the release of antimicrobial peptides (AMP) by Paneth cells and maintain gut health.

Through microbial fermentation of complex carbohydrates in the colon, SCFAs are synthesized as essential energy substrates for IEC. Butyrate, acetate, and propionate - the primary SCFAs - modulate host immune responses via PRRs expressed in IEC and are characterized by anti-inflammatory properties (Figure 3) [66].

Colon cells metabolize SCFAs to produce adenosine triphosphate through β-oxidation and citric acid cycle. Butyrate, in particular, is the preferred energy source [67]. Besides providing energy, butyrate can also regulate intestinal immunity and barrier by triggering adenosine 5′-monophosphate-activated protein kinase (AMPK). In addition, butyrate can exert antimicrobial effects by modifying the metabolism of macrophages and upregulating the expression of AMPs through inhibiting the activity of histone deacetylase 3 [68]. In piglets, the modulatory role of butyrate in balancing apoptosis and proliferation, as well as stimulating intestinal development, has been demonstrated [69]. Intestinal flora-derived SCFAs can provide energy for pregnant sows [70], regulate the host energy homeostasis through G protein-coupled receptors (GPR)41 and GPR43 in intestine, nervous system and embryo, and exert anti-inflammatory effects [71], which ultimately benefit sows and piglets. Of course, the intestine’s anaerobic environment contributes to the synthesis of SCFAs as well. The anaerobic environment of intestine can enrich obligate anaerobic bacteria, such as *Lachnospiraceae* NK4A136, which may generate a significant amount of SCFAs [72]. Similarly, SCFAs can switch colon cells’ energy metabolism to β-oxidation through PPAR-γ signal [73], so as to maintain the anoxic environment and form a virtuous circle. SCFAs mediate inflammatory processes by interacting with related receptors, and mucosal immunity and SCFAs interact in a complex manner, which should be further studied.

More and more studies show that the gut microbiota and its metabolites have vital effects on physiology and immune function of the host. Targeted manipulation of the gut microbiota and its metabolites is expected to enhance piglet health, although the full extent of their impact remains incompletely understood. Studies conducted both *in vivo* and *in vitro* have offered valuable insights into the overall effects of gut flora and metabolites on piglet growth and health. Additionally, the use of omics technology can enhance our understanding of these complex interactions.

## MEASURES TO REGULATE PIGLETS HEALTH

### Probiotics

In the pig industry, probiotics have been widely utilized to regulate the immune system, optimize gut ecology, and promote health due to their potential to compete with pathogens for sites of adhesion and modulate host immune responses [74,75].

Oxidative stress in the intestine often leads to intestinal inflammation as well as barrier damage, accompanied by microbiota disruption, severely limiting the pig industry. Due to their antioxidant properties, various strains of lactic acid bacteria have been extensively used in pig farming. For weaned piglets, lactic acid bacteria supplementation increases the relative abundances of *Lactobacillus* and *Bifidobacterium*, decreases the relative abundance of *E. coli*, and increases the SCFAs content in the intestinal [76,77]. Besides these functions, probiotics can promote the healthy growth of the host by regulating the immune system and intestinal symbiotic bacteria. For instance, probiotics can increase the production of sIgA, stimulate the secretion of intestinal mucin and AMPs, and promote the proliferation and differentiation of intestinal immune cells [78]. For weaned piglets, *Bifidobacterium* has the function of improving intestinal health and immunity. Supplementation of *Bifidobacterium* AH1206 resulted in a dose-dependent linear rise in IL-10 expression. Th2 and T-regulatory type 1 cells produce IL-10, which is necessary for antibody production and immune tolerance [79]. Providing *Bifidobacterium animalis* subsp. *Lactis* to weaned piglets is helpful to reduce piglet diarrhea, and raise the relative abundances of beneficial bacteria (*Streptococcus*, *Coprococcus*, and *Oscillibacter*) in the gut [80]. *Lactobacillus salivarius* can regulate piglet immunological responses by regulating cytokines. It can also stimulate the production of antioxidant enzymes like superoxide dismutase, glutathione peroxidase 4, and catalase through the Nrf2/HO-1 pathway [81]. In addition, *Lactobacillus gasseri*, *Lactobacillus reuteri*, *Lactobacillus acidophilus*, and *Lactobacillus casei* have also been shown to be effective in relieving piglets diarrhea [82].

The observed enhancement in growth performance of piglets administered probiotics can be ascribed to the positive impacts of probiotics on gut health, including reduction of pathogen colonisation in the gut, regulation symbiotic bacteria, enhancement of gut barrier function and stimulation of the mucosal immune system. However, it is crucial to underscore that the dosage, type, mixing ratios, and duration of treatment of probiotic preparations remain to be determined. In addition, there is a need to address the potential risk of probiotic strains introducing resistance genes into the microbial ecosystem.

### Amino acids

Dietary proteins and amino acids (AAs) are key substrates for intestinal microbial fermentation in pigs. Furthermore, AAs can be employed as nitrogen sources to facilitate the proliferation of intestinal microflora [83]. In the distal intestine, AAs usually have three possible metabolic pathways: (i) excreted with feces; (ii) utilized for the synthesis of protein by gut microorganisms; (iii) metabolized into other substances by gut microorganisms. Gut microbiota is the link between dietary AAs and host immunity. Microbial community composition is determined by dietary AAs, which consequently regulates the metabolism of AAs, with both directly or indirectly affecting the host’s immunity [84].

For instance, dietary L-Asparagine (ASN) is a nonessential AA that can reduce LPS-induced intestinal dysfunction in piglets through the regulation of the corticotrophin-releasing factor (CRF)/CRF receptor signaling pathway. Moreover, ASN supplementation raised the amount of IEL while reversing the inflammatory response induced by LPS stress [85]. According to existing studies, AAs affect gut microbes in various ways. Dietary AAs promote the production of β-defensins and other AMPs (endogenous cationic peptides) in the gut, thus suppressing harmful microorganisms growth [86]. Along with AAs, the health of the gut has also been associated with metabolites of AAs. Trp is an essential aromatic AA that the body cannot synthesise. Most of the dietary Trp absorbed by the body is participating in the synthesis of proteins, and the rest of the Trp produces indole, indole derivatives, kynurenic acid, 5-HT and other active substances through various metabolic pathways [87]. Trp can decrease intestinal inflammation induced by Enterotoxigenic *E. coli* (ETEC) K88 through the calcium-sensing receptors (CaSR)/Ras-related C3 botulinum toxin substrate 1/Phospholipase C γ 1 signaling pathway [88]. Insufficient Trp in the diet reduces the absorption and utilisation of protein in pigs, leading to low immunity and increased susceptibility to disease. Dietary Trp supplementation may promote intestinal health by improving antioxidant status, alleviating inflammation in the piglets’ gut after LPS attack, and inhibiting genes involved in cellular focal death [89]. To yet, the effect of dietary Trp on focal death has not been well studied, and its precise mechanisms must be examined further. Within the intestinal microenvironment, host defence mechanisms mediated by AMPs play a pivotal role in pathogen exclusion and maintenance of mucosal ecological equilibrium. Through CaSR-Trp metabolism, Trp can promote the generation of PBD1 and PBD2 while activating the CaSR-AMPK pathway to reduce intestinal inflammation [90]. The immune system has long been a therapeutic target for inflammatory or infectious diseases, and it is feasible to modulate intestinal immunity through dietary Trp supplementation to promote gut health in piglets. Gamma-aminobutyric acid (GABA), a neurotransmitter, also plays an important function in the immune system. A diet supplemented with GABA may ameliorate weaning stress effects on piglets by regulating their endocrine functions [91]. In ETEC-induced intestinal inflammation in piglets, GABA derived from the gut microflora can also increase the expression of IL-17 by activating the signal transduction of mTOR complex 1-ribosomal S6 kinase 1 [92]. Adding GABA can significantly reduce the damage caused by ETEC. Additionally, it can improve intestinal mucosal immunity by promoting the secretion of sIgA in the jejunum. This effect may be connected to T cell-dependent pathways and changes in the structure and metabolic in the gut microbiota [93].

More and more researches suggest that AAs serve not only as constituents of muscle protein, but also as functional substances. Due to their various roles in energy, functional molecular precursors, signaling molecules and microbiota modulators, AAs have the potential to strengthen gut health in monogastric animals by protecting the intestinal barrier integrity, adjusting the balance of microbiota, and bolstering immunity. Further investigation is necessary to optimize the utilization of AAs’ functions while minimizing the required supplement dosage of AAs.

### Faecal microbiota transplantation

Faecal microbiota transplantation (FMT) involves the transplantation of faecal suspensions from a healthy donor to a recipient as a therapeutic intervention for various medical conditions, such as intestinal immunodeficiency, intestinal allergies, metabolic diseases, inflammatory bowel disease, and *Clostridium difficile* infections. Additionally, FMT has garnered attention in the pig industry for its potential benefits [94,95]. After weaning, the variety of the microbiota in the gut decreases, increasing vulnerability to post-weaning diarrhea and intestinal infection [96]. As a special treatment method, FMT can regulate the intestinal health of piglets by rebuilding intestinal microecology.

Jinhua pigs, a renowned indigenous pig breed in China, exhibit heightened resistance to *E. coli* infection. The transplantation of the Jinhua pig faeces suspension into Duroc× Landrace×Yorkshire piglets contributed to an increase in Prevotellaceae, Firmicutes, *Ruminococcus*, and other microorganisms in the recipient piglets’ colons, as well as a reduction in diarrhea. In addition, the morphology and integrity of the gut were improved with a rise in goblet cells, MUC2 protein and immune-related receptors [97]. As a critical member of the IL-10 cytokine family, IL-22 is predominantly produced by ILC3 and mediates intestinal barrier immunity and systemic immunological regulation [98]. Trp metabolism in recipient piglets produces indole derivatives, which can help preserve intestinal integrity by generating IL-22 that is dependent on the AHR [64]. Piglets of the Duroc×Landrace×Yorkshire breed infected with *E. coli* K88 were given a transplant with the faecal microbial suspension of Jinhua pigs. Study outcomes demonstrated that FMT reduced LPS-induced intestinal inflammation and modulated the Trp metabolic function of the microbial communities in recipient piglets’ colons. The number of beneficial bacteria such as *Lactobacillus* and *Succinivibrio* increased in infected piglets after FMT, while the number of *Enterobacteriaceae* and *Proteobacteria* decreased. Additionally, the intestinal permeability of recipient piglets was decreased, and the expression of mucins and tight junction proteins in the intestinal tract were enhanced, which is conducive to reducing piglets’ diarrhea. The intervention of exogenous faecal microbiota increased Forkhead box O-mediated protective autophagy in the recipient intestinal mucosa, alleviating IEC damage [99]. It was shown that FMT can protect the integrity of the intestinal barrier by triggering mucosal protective autophagy. Tibetan pigs, which have excellent disease resistance, have become a popular option for donors. Oral administration of faecal microbiota from healthy Tibetan pigs resulted in significant reductions in diarrhea rates and tissue damage scores in recipient piglets. In addition, FMT treated markedly an increase in colon length, improved the microbial flora, and could regulate the immune function of the recipient piglets. Moreover, FMT treatment could reduce piglets’ diarrhea, downregulate the TLR signaling pathway and inducible nitric oxide synthase gene expression, and improve the integrity of the intestinal via controlling the composition of the intestinal microbiota [100].

These results suggest that FMT can remodel the pig gut microbiota. However, the precise mechanism and optimal scheme have not been thoroughly studied, and there is considerable uncertainty. Using FMT as a strategy to enhance pig health, increase feed efficiency, or prevent or treat illnesses is still in the beginning stages. Due to the scarcity of data, it is difficult to conclude definitively on the effectiveness of FMT in piglets. There are obvious biological safety problems in FMT, and it is difficult to screen all possible harmful microorganisms in donors with current technology. In addition, FMT still faces many challenges, such as obtaining high-quality samples, reducing the risk of infection and disease transmission, and ensuring the stability and long-term effect of FMT. Especially in the field of intensive pig production, a product with low cost and high quality is needed. However, considering that there have been many successful cases of FMT in human beings, it is believed that remarkable achievements can be made in the pig industry.

## CONCLUSION AND PERSPECTIVES

The weaning of piglets is a critical step in their growth and development. The gut microbiome and immune system of piglets are immature at this stage, and piglets are sensitive to sickness and have poor defences against outside influences. Intestinal immunity, as a line of immune system defence, is very important for the control and avoidance of diseases. The gut microbes are indispensable to the development of the intestinal mucosal immune system, many of which can also have an influence on the differentiation of immune cells. Although research on host immune-microbiota interactions is continuously evolving and the precise mechanisms still require further investigation, it is evident that gut microorganisms and their metabolites possess significant potential to regulate host health. These studies will help provide effective strategies for managing and restoring gut homeostasis to improve productivity, reduce stress, prevent disease, and promote healthy development of the pig industry in the future.
